# Strategies to enhance management of HER2-positive breast cancer in the elderly: an expert consensus perspective

**DOI:** 10.1007/s12094-024-03838-1

**Published:** 2025-01-10

**Authors:** Sonia del Barco, Almudena Cotes-Sanchís, Mercedes Cavanagh, Regina Gironés-Sarrió, Borja López de San Vicente, Elena Galve-Calvo, Sonia Servitja

**Affiliations:** 1 Department of Medical Oncology, Spanish Society of Medical Oncology (SEOM) Oncogeriatrics Section, Catalan Institute of Oncology (ICO), Doctor Josep, Trueta University Hospital, Avinguda de França, S/N, 17007 Girona, Spain; 2 Medical Oncology Department Alicante, Spanish Society of Medical Oncology (SEOM) Oncogeriatrics Section, Elda Virgen de la Salud General University Hospital, Elda, Spain; 3 Medical Oncology Department, Faculty of Biomedical and Health Sciences, Spanish Society of Medical Oncology (SEOM) Oncogeriatrics Section, Getafe University Hospital, Madrid European University, Madrid, Spain; 4 Medical Oncology Department, Spanish Society of Medical Oncology (SEOM) Oncogeriatrics Section, La Fe University and Polytechnic Hospital, Valencia, Spain; 5 Medical Oncology Department, Spanish Society of Medical Oncology (SEOM) Oncogeriatrics Section, Basurto University Hospital, Bilbao, Spain; 6 Medical Oncology Department, Spanish Group of Study, Treatment and Other Experimental Strategies in Solid Tumours (SOLTI), Basurto University Hospital, Bilbao, Spain; 7 Medical Oncology Department, Hospital del Mar, Parc de Salut Mar, Spanish Group for Breast Cancer Research (GEICAM), Barcelona, Spain

**Keywords:** Breast cancer, Elderly patient, Geriatric assessment, HER2-positive, Toxicity

## Abstract

Therapeutic decision-making for older patients with human epidermal growth factor receptor 2 (HER2)-positive breast cancer highlights the importance of a comprehensive geriatric assessment (CGA). This assessment considers the functional status, comorbidities, and relevant conditions of the patient, and allows for an estimation of life expectancy, but it does not facilitate individualized treatment plans. There are also other challenges to consider related to the cardiac toxicity of the treatments and the under-representation of older patients in clinical trials. The Oncogeriatrics Section of the Spanish Society of Medical Oncology (Sociedad Española de Oncología Médica, SEOM), the Spanish Group for Breast Cancer Research (Grupo Español de Investigación en Cáncer de Mama, GEICAM) and the Spanish Group of Study, Treatment and other Experimental Strategies in Solid Tumours (Grupo Español de Estudio, Tratamiento y otras Estrategias Experimentales en Tumores Sólidos, SOLTI) have gathered an expert committee to evaluate the scientific evidence on the management of older patients with HER2-positive breast cancer and to establish recommendations based on a comprehensive review of the existing literature. These recommendations underscore the importance of individualizing treatment plans based on the patient's physical status and tolerability to maximize efficacy while minimizing toxicity. Emphasis is placed on adapting neoadjuvant and adjuvant therapies according to geriatric assessment and specific patient needs. A careful selection of treatment schedules for advanced stages is needed to improve survival and quality of life, assuming that scientific evidence in this age group is limited.

## Introduction

As life expectancy continues to increase, cancer cases in the elderly population are expected to rise, given that age is one of the main risk factors for developing neoplasms [1]. Despite the absence of a consensus on the definition of an older patient, there is a growing tendency to consider that the age cut-off should be set at 70 years [2]. A distinct biological profile is observed in older breast cancer patients, characterized by a higher tendency to positive hormonal receptors and a lower expression of the human epidermal growth factor receptor 2 (HER2) [2].

There is some concern regarding the management of elderly patients with HER2-positive tumors, mainly due to the cardiac toxicity risk associated with the treatment and the cardiac comorbidities associated with aging [3]. Furthermore, there is limited scientific evidence on this subject, due to the under-representation of older patients expressing HER2 in clinical trials [4, 5]. The complex interplay of comorbidities and increased susceptibility to treatment toxicity due to age-related physiological changes makes therapeutic decision-making particularly challenging. For instance, cardiovascular disease prevalence in elderly patients can significantly impact treatment options, given the cardiac risks associated with HER2-targeted therapies [6]. Recent literature emphasizes the need for treatment frameworks that balance effectiveness with safety, particularly for elderly patients undergoing neoadjuvant or adjuvant therapy [7–9].

This revised consensus paper aims to evaluate the existing scientific evidence regarding the management of the elderly population (≥ 70 years) with HER2-positive breast cancer. To this end, the Oncogeriatrics Section of the Spanish Society of Medical Oncology (Sociedad Española de Oncología Médica, SEOM), the Spanish Group for Breast Cancer Research (Grupo Español de Investigación en Cáncer de Mama, GEICAM), and the Spanish Group of Study, Treatment and other Experimental Strategies in Solid Tumours (Grupo Español de Estudio, Tratamiento y otras Estrategias Experimentales en Tumores Sólidos, SOLTI) have developed optimal management recommendations based on a comprehensive review of the available literature.

The consensus was reached through a structured review and discussion process conducted by all authors, who collectively examined available evidence on HER2-positive breast cancer in the elderly. Studies published in peer-reviewed journals were reviewed and the Agency for Healthcare Research and Quality (https://www.ahrq.gov/) was used to assign levels of evidence and grades of recommendation. Recommendations were formed using an iterative process in which the expert committee assessed and integrated relevant findings from clinical trials, real-world studies, and expert opinion.

## Comprehensive geriatric assessment of HER2-positive breast cancer patients

Comprehensive geriatric assessment (CGA) is considered essential to accurately estimate life expectancy and guide decision-making on the best possible treatment in the field of geriatric oncology. For women with breast cancer, advanced age has proven to be an important risk factor for increased mortality. The comorbidity associated with the age of these patients has a significant impact on the prognosis of the disease [7, 9].

The CGA covers several domains: functional status, comorbidities, polypharmacy, depressive symptoms, cognitive status, psychosocial distress, nutritional status, and socioeconomic support. The European Society of Breast Cancer Specialists (EUSOMA) and the International Society of Geriatric Oncology (SIOG) recommend CGA to be used in the management of all older breast cancer patients [8, 10].

For each type of assessment, the most commonly used scales to date are (i) the Barthel index and Lawton–Brody scale for functional status; (ii) the Pfeiffer questionnaire for cognitive function; (iii) the Mini Nutritional Assessment MNA-SF for nutritional status; (iv) the Yesavage scale for psychological and mood assessment; (v) the Gijón scale for the evaluation of socio-familial support; (vi) the Cumulative Illness Rating Scale-Geriatric (CIRS-G) and Charlson index for the assessment of comorbidities; (vii) list of drugs used; and viii) geriatric syndromes, such as urinary incontinence, insomnia, domestic abuse, presbycusis, etc. These latter may be detected by activities of daily living (ADLs) and instrumental activities of daily living (IADLs). The first ones cover basic self-care skills and the maintenance of independence (independent mobility, transfer, continence, and feeding), while IADLs are more complex and require more autonomy (decision-making, problem-solving, money management, use of public transport, medication intake, and meal preparation). Comorbidities refer to one or more disorders in addition to the specific cancer, which become increasingly prevalent with age and are associated with poorer cancer patient outcomes [11].

Within the scales used for CGA, there are independent predictors of mortality in breast cancer patients, such as impaired functional status, comorbidity, and depression [12]. In summary, an accurate CGA detects vulnerabilities in all domains to personalize treatment and care according to the needs of the patient, but the classification is neither easy nor “rigid over time” as can be seen in Table 1.

**Table 1:** Patient´s classification based on CGA [2, 9]

Healthy	Vulnerable	Frail
Tolerates standard treatment and gains benefit equivalent to that of younger patients (< 65 years)	Less tolerant to standard treatment, but amenable to intervention on modifiable areas of vulnerability	Presents impairment in multiple physiological systems, often but not exclusively age-related. The patient is in a pre-disability state that coexists with disability and chronic diseases
Performs ADLs and IADLs independently No signs of malnutrition G8 ≥ 15 Grade ≤ 2 in all CIRS-G categories	Performs ADLs independently, but shows slowness and fatigue Dependent for IADLs Loss of 5–10% of body weight, with signs of malnutrition G8 ≤ 14 At least one grade 3 in CIRS-G categories Has a history of falls in the last 6 months	Indicates severe malnutrition Comorbidities usually decompensated G8 ≤ 14 At least one grade 4 in CIRS-G categories

ADLs: activities of daily living; CGA: comprehensive geriatric assessment; CIRS-G: Cumulative Illness Rating Scale-Geriatric; G8: Geriatric 8 Screening Tool; IADLs: instrumental activities of daily living

**Fig. 1:**
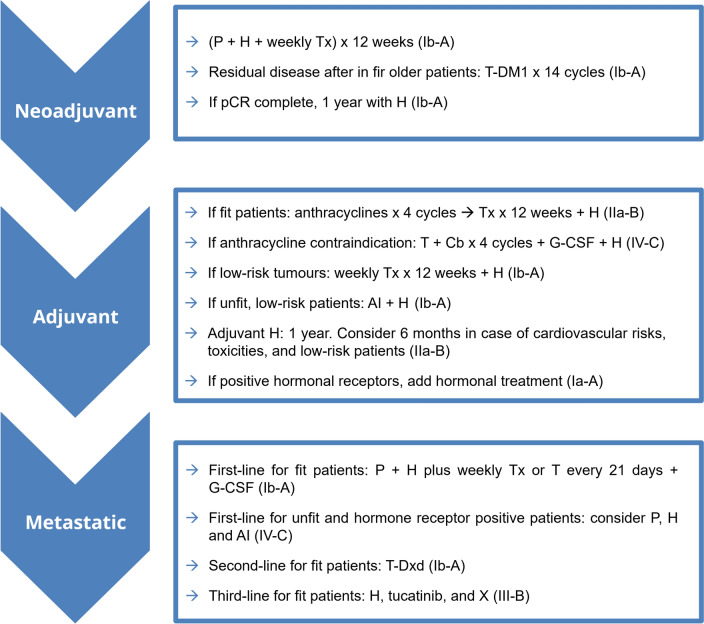
Therapeutic options and recommendations for patients ≥ 70 years old with HER2-positive breast cancer. Neoadjuvant treatment: (i) same recommendations as in the adjuvant context and for younger patients; (ii) more evidence of less toxic regimens is needed. Patients with HER2-positive metastatic breast cancer should receive anti-HER2 therapy with cardiological assessment if congestive heart failure or LVEF ≤ 50%. The following factors should be considered: (i) risk of relapse; (ii) life expectancy (evaluated through a geriatric assessment); (iii) tolerability (including cardiac tolerability); (iv) Patient preferences. AI: aromatase inhibitor; AUC: area under the curve; Cb: carboplatin; D: day; G-CSF: Granulocyte Colony-Stimulating Factor; H: trastuzumab (Herceptin®); LVEF: left ventricular ejection fraction; N0: node negative; P: pertuzumab; pCR: pathological complete response; T: docetaxel (Taxotere®); T-DM1: trastuzumab emtansine; T-Dxd: trastuzumab deruxtecan; Tx: paclitaxel (Taxol®); X: capecitabine (Xeloda®)

As discussed throughout this article, the therapeutic approach to HER2-positive breast cancer in older patients must be carefully personalized, considering the diversity of physical conditions, comorbidities, treatment tolerance and the patient’s preferences regarding their quality of life (Fig. 1).

## Treatment of the older patient with HER2-positive early breast cancer

### Neoadjuvant treatment

Healthy older patients should be treated with the same neoadjuvant treatment as younger patients since the high probability of pathological complete response (pCR) is considered a prognostic factor for survival and may avoid lymphadenectomies and thus reduce comorbidity (Ia-A). A meta-analysis conducted by a German research group revealed that older women who received anti-HER2 therapy exhibited a higher rate of pCR compared to those who did not receive such treatment [13].

Although adjuvant trastuzumab is beneficial across all age subgroups, neoadjuvant anti-HER2 strategies are poorly studied in patients > 65 years. However, it is now widely accepted that trastuzumab is effective and well-tolerated in these patients, remaining the primary treatment for anti-HER2 therapy (Ib-A). The NeoSphere and TRYPHAENA studies, which led to the approval of dual anti-HER2 blockade (trastuzumab–pertuzumab) in combination with chemotherapy, included patients up to 80 years, although the median age was 50 years (Table 2). The treatment followed in these studies increased the pCR rate (46–66%) with a low incidence of left ventricular dysfunction (2–13%), but the studies did not include age-specific data. Therefore, while efficacy can be extrapolated, it is not possible to determine whether older patients experienced greater toxicity [14, 15]. Similarly, data by age subgroups were not published in the WSG-ADAPT HER2 + /HR- study, where the median age of the included patients was 51 years and the combination of weekly paclitaxel with trastuzumab–pertuzumab achieved a pCR rate of 91% [16]. Unfortunately, the TRAIN-2 study did not include patients older than 56 years. This study demonstrated that omitting anthracyclines in the neoadjuvant setting is a much less toxic option, with similar high pCR rates in both treatment groups [17].

**Table 2:** Neoadjuvant and adjuvant treatment for HER2-positive early breast cancer

Study	Patients	Treatment	Results
*Neoadjuvant treatment*			
TRYPHAENA Phase II NCT00976989 [14]	n = 225 50 yr (27–81)	A: FEC + H + P × 3 → T + H + P × 3 B: FEC × 3 → T + H + P × 3 C: TCbH + P × 6	A: pCR: 62% B: pCR: 57% C: pCR: 66%
NeoSphere Phase II NCT00545688 [15]	n = 417 50 yr (22–80)	A: H + T B: PH + T C: PH D: P + T	A: pCR: 29% B: pCR: 46%; *p* = 0.014 C: pCR: 17% D: pCR: 24%
WSG-ADAPT Phase II NCT01779206 [16]	n = 160 51 yr (28–78)	A: PH + Tx B: PH	A: pCR: 91% B: pCR: 36%
The Royal Marsden Experience [18]	n = 789 50 yr (24–81) 11.5% ≥ 65 yr	A: CT + PH B: CT + H	< 35 yr: pCR: 38% 35-64 yr: pCR: 33% ≥ 65 yr: pCR: 32%; *p* = 0.724
Choi et al. [19]	n = 19 55 yr (44–78) 5% ≥ 75 yr	PH + Tx	50–74 yr: pCR: 70% ≥ 75 yr: pCR: 100%
Williams et al. [20]	n = 651 12% ≥ 70 yr	CT + PH	39–50 yr: pCR: 42% ≥ 70 yr: pCR: 43%
*Adjuvant treatment*			
Jones et al NCT00493649 [21]	n = 493	TC + H	2-yr DFS: 98% 2-yr OS: 99%
Brollo et al. [22]	n = 1084 100% > 60 yr	A: CT + H B: CT	A *vs.* B: Reduced RR by 47%; HR 0.53; 95% CI 0.36–0.77
Tolaney et al NCT00542451 [23]	n = 406 55 yr (24–85)	Tx + H	10-yr DFS: 99% 10-yr OS: 94%
PHARE Phase III NCT00381901 [24]	n = 3384	A: H for 6 months B: H for 12 months	A *vs*. B 4-yr DFS: 76% *vs*. 79%
PERSEPHONE Phase III NCT00712140 [25]	n = 2045	A: H for 6 months B: H for 12 months	A *vs*. B 4-yr DFS: 89% *vs.* 90%
Sawaki et al NCT01104935 [26]	n = 275 74 yr (70–80)	A: CT + H B: H	A *vs*. B 3-yr DFS: 94% *vs*. 90% 3-yr RFS: 93% *vs*. 92%
APHINITY Phase III NCT01358877 [27]	n = 4805 13% > 65 yr	A: CT + PH B: CT + H + Placebo	A *vs*. B 3-yr IDFS: 94% *vs.* 93%
ExteNET Phase III NCT00878709 [28]	n = 2840 ≤ 60 yr	A: Neratinib B: Placebo	A *vs*. B 5-yr IDFS: 90% *vs.* 88% 8-yr OS: 90% vs. 90%
KATHERINE Phase III NCT01772472 [29]	n = 1486 < 10% > 65 yr	A: T-DM1 B: H	A *vs*. B 3-yr IDFS: 88% *vs*. 77%

C: cyclophosphamide; Cb: carboplatin; CI: confidence interval; CT: chemotherapy; DFS: disease-free survival; FEC: 5-fluorouracil, epirubicin, and cyclophosphamide; H: trastuzumab (Herceptin®); HR: hazard ratio; IDFS: invasive disease-free survival; OS: overall survival; P: pertuzumab; pCR: pathological complete response; RFS: recurrence-free survival; RR: recurrence risk; T: docetaxel (Taxotere®); T-DM1: trastuzumab emtansine; Tx: paclitaxel (Taxol®); yr: years

In the absence of specific data from registry studies, some evidence in patients ≥ 65 years has been obtained through studies with real-world data (III-B): (i) in a retrospective cohort of patients at the Royal Marsden hospital treated with chemotherapy and double blockade or trastuzumab monotherapy [18], patients aged ≥ 65 years exhibited a pCR rate of 32%, compared to 38% in patients < 35 years and 33% in patients aged 35–64 years (*p* = 0.724); (ii) a small case series study conducted in Australia included 19 patients treated with weekly paclitaxel and trastuzumab–pertuzumab [19], showing a pCR rate of 70% in patients aged 50–74 years and a 100% rate in patients ≥ 75 years; (iii) in another real-world study on the safety of neoadjuvant treatment in women aged ≥ 65 years [30], 45 patients with a median age of 67 years treated with carboplatin–docetaxel plus trastuzumab–pertuzumab or trastuzumab alone showed similar toxicities between the two treatment regimens; and (iv) in a retrospective series from Memorial Sloan Kettering Cancer Centre, in which 651 women received neoadjuvant treatment [20], of whom 30 (12%) were ≥ 70 years with HER2-positive tumors and were treated with chemotherapy and trastuzumab–pertuzumab, the pCR rate was similar between patients ≥ 70 years and those aged 39–50 years, although a higher proportion of patients in the former group did not complete the planned treatment schedule.

Therefore, the available data summarized in Table 2 suggest that pertuzumab is effective and safe in the elderly population, although there is an increased risk of developing diarrhea, dehydration, and acute renal failure in these patients, especially if it is given concomitantly with docetaxel [31].

Based on the evidence previously described, we recommend the use of dual anti-HER2 blockade with trastuzumab and pertuzumab in fit elderly patients (Ib-A and IIa-B, respectively), with the intention of achieve a higher pCR. As there are no specific neoadjuvant studies in patients ≥ 70 years of age, the administration of 12 cycles of concomitant weekly paclitaxel would be advisable as long as grade > 2 toxicities do not appear.

### Adjuvant treatment

In patients with HER2-positive early (stages I–III) breast cancer, the combination of trastuzumab with chemotherapy after surgery has been shown to reduce the recurrence risk (RR) (0.66; 95% confidence interval [CI] 0.62–0.71; *p* < 0.001) and breast cancer mortality (0.67; 95% CI 0.61–0.73; *p* < 0.001); thus, it has become the standard of care for these patients. These improvements were independent of the chemotherapy regimen used and the age of the patients (although less than 30% of patients > 70 years were included in these studies). Therefore, treatment in elderly patients should be based on individual toxicity and RR (Ia-A) [21, 32].

A meta-analysis of the main adjuvant studies included 1084 patients > 60 years with HER2-positive early breast cancer and showed that the addition of trastuzumab to chemotherapy reduced the RR by 47% (hazard ratio [HR] 0.53, 95% CI 0.36–0.77), with 5% of cardiac events (95% CI 4–7%) [22]. Different strategies to reduce the toxicity of adjuvant treatment in older patients include (i) the use of anthracycline-free or less toxic regimens, such as weekly paclitaxel with trastuzumab, which showed a 10-year overall survival (OS) of 94% and a 10-year breast cancer-specific survival of 99% in a phase II study in patients with tumors < 3 cm (Ib-A) [23]; (ii) a reduction in trastuzumab treatment from 12 to 6 months could be an option for frail elderly women or those with cardiovascular comorbidity (IIa-B) [24, 25]; (iii) although less toxic, trastuzumab monotherapy should not be recommended in older patients following a Japanese study of 275 elderly patients aged 70–80 years (median age of 74 years), which failed to demonstrate non-inferiority of adjuvant trastuzumab alone versus trastuzumab in combination with chemotherapy [26].

There are also treatment intensification strategies for patients at a higher risk of relapse: (i) double blockade with pertuzumab and trastuzumab plus chemotherapy showed an improvement in recurrence-free survival and no significant changes in OS in the APHINITY study, which only included 13% of patients > 65 years. This treatment regimen also showed increased toxicity in the form of diarrhea and neutropenia, so its administration should be carefully evaluated in the elderly population with high-risk tumors [27]; (ii) although the ExteNET study did not include patients aged ≥ 60 years, the benefit of extending anti-HER2 therapy with neratinib in older patients should be assessed with caution. This is due to the narrow therapeutic index of the drug and the high risk of severe diarrhea as an adverse effect, which may be particularly problematic in this group of patients [28]; (iii) in the KATHERINE study, which included patients with residual disease following neoadjuvant treatment, trastuzumab emtansine (T-DM1) reduced the risk of recurrence and mortality (HR 0.50; 95% CI 0.39–0.64; *p* < 0.001) although only 16% of all patients were ≥ 65 years [29].

Based on previous findings, the administration of adjuvant trastuzumab along with chemotherapy is recommended in elderly patients (Ia-A), however other therapeutic options with less toxicity could be administered as well (IIb-A). If after neoadjuvant treatment, pCR was not achieved, T-DM1 is indicated for fit patients (Ib-A).

## Treatment of the older patient with HER2-positive advanced breast cancer

### First-line treatment

The strategy for first-line treatment is established after considering the previous treatment history of the patient (either de novo disease or relapse after adjuvant treatment with or without neoadjuvant therapy), hormone receptor status, and a detailed CGA that considers the patient's frailty and personal preferences. The basis of first-line therapy relies on the combination of dual HER2 blockade and chemotherapy (Ib-A). The use of this treatment is supported by the results of the CLEOPATRA clinical trial [33], which demonstrated a significant increase in progression-free survival (PFS) (18.7 vs. 12.4 months; HR 0.68; 95% CI 0.58–0.80; *p* < 0.001) and OS (56.5 vs. 40.8 months; HR 0.68; 95% CI 0.56–0.84; *p* < 0.001) with the addition of pertuzumab to docetaxel and trastuzumab. Although the representation of older patients in this study was limited, with only 127 (16%) patients ≥ 65 years out of 808 patients, the subgroup analyses suggested that the benefit in terms of PFS is consistent in older patients. However, the toxicity profile was slightly different, showing an increased incidence of diarrhea, asthenia, hyperoxia, and vomiting, which highlights the importance of patient selection and close patient monitoring.

For physically fit patients, the recommended combination includes double anti-HER2 blockade together with paclitaxel, given its superior tolerability profile (IIa-B) [34]. Following an initial period of induction chemotherapy, patients may then proceed to maintenance treatment with double HER2 blockade plus hormonal therapy in the case of hormone receptor-positive patients, thus reducing the potential risk for additional toxicities associated with chemotherapy (IIa-B). Nevertheless, tolerance to taxanes may be suboptimal in vulnerable individuals. The combination of hormonal therapy with anti-HER2 agents has emerged as a valuable treatment option for hormone receptor-positive tumors with minimally symptomatic disease as demonstrated in the PERTAIN trial (Ib-A) [35]. In cases of rapidly progressive or symptomatic disease, combination with chemotherapy is preferred (IV-C). However, in patients who are too frail or who have contraindications to taxanes, studies, such as HERNATA [36], VELVET [37], and EORTC 75111–10,114 [10], have shown good tolerability and efficacy of treatment regimens combining vinorelbine or metronomic cyclophosphamide with trastuzumab or double HER2 blockade monotherapy (Ib-A/IIb-B).

In the context of relapse following neoadjuvant and/or adjuvant treatment, guidelines recommend several treatment alternatives depending on residual toxicity and time to relapse (> 6 or < 6-month treatment-free interval), including reuse of trastuzumab with or without pertuzumab plus a taxane or the progression to newer lines of treatment, such as trastuzumab deruxtecan (T-DXd) [38].

Based on these results, we recommend the use of dual HER2 blockade and taxane-based chemotherapy for fit older patients (Ib-A) and the use of alternative regimens in more vulnerable patients (IIb-B).

### Successive lines of treatment

#### 4.2.1 Trastuzumab deruxtecan

The DESTINY-Breast03 study revealed the superiority of T-DXd over T-DM1 in patients with HER2-positive metastatic breast cancer previously treated with anti-HER2 therapies, positioning it as a standard for second-line treatment. Among the 524 randomized patients, 261 received T-DXd of which 57 were aged ≥ 65 years (22%) and 8 were aged ≥ 75 years (3%). About 50% of patients in these groups had visceral involvement, and 10 had brain metastases [39]. A comparison of efficacy between patients younger and older than 65 years revealed no significant difference in the median PFS (30 months *vs.* 25 months) and the objective response rate (ORR) (79% *vs.* 78%) [40]. The DESTINY-Breast12 trial, with primary results recently presented at the European Society of Medical Oncology (ESMO) 2024 congress, demonstrated that T-DXd showed significant and durable efficacy in patients with metastatic HER2-positive breast cancer and brain metastases. The study included 263 patients (median age of 52 years) with these metastases, exhibiting a median PFS of 17.3 months and a 12-month PFS rate of 62%. These results support the use of T-DXd in older patients although specific data for those > 70 years was not available [41].

An analysis presented at the 2023 American Society of Clinical Oncology (ASCO) meeting included an evaluation of the efficacy and safety of T-DXd in patients younger and older than 65 years, covering data from the DESTINY-Breast01, 02, and 03 studies. In total, the analysis included 673 women < 65 years, with 178 ≥ 65 years and 34 ≥ 75 years treated with T-DXd. This analysis confirmed similar efficacy across age subgroups, with a comparable relative dose intensity and an acceptable safety profile. However, compared to patients < 65 years, those aged ≥ 65 years exhibited a higher rate of treatment discontinuations, interstitial lung diseases or pneumonitis (12% *vs.* 18%) and grade ≥ 3 drug-related adverse events, such as nausea, fatigue and hematologic toxicities, including neutropenia and anemia (44% *vs.* 54%). Although the efficacy of T-DXd was maintained in all age subgroups and its safety profile was generally acceptable, it is crucial to consider comorbidities and quality of life, especially in older patients [39].

Given the high emetic risk of T-DXd, it is recommended to use a triple antiemetic prophylaxis regimen, including a 5-hydroxytryptamine-3 receptor antagonist (5-HT3RA), dexamethasone (DEX), and a neurokinin-1 receptor antagonist (NK1RA) as support by previous studies [42]. Additionally, olanzapine was found to be effective in controlling nausea, further enhancing the management of chemotherapy-induced nausea and vomiting [43]. Also, interstitial lung disease and pneumonitis are significant risks associated with T-DXd. Thus, patients should be promptly informed to report any respiratory symptoms, as early recognition is essential for management. Multidisciplinary guidelines are available to support monitoring, diagnosis, and treatment, helping reduce risks of progression and improve outcomes [44].

Based on the evidence described, we recommend the use of T-DXd in elderly patients (Ib-A) due to its efficacy in this population when other previous treatments proved to be unsuccessful.

#### 4.2.2 Tucatinib

Tucatinib in combination with trastuzumab and capecitabine offers significant benefits in PFS and OS in patients with HER2-positive metastatic breast cancer who have previously been treated with trastuzumab, pertuzumab, and T-DM1 compared to placebo, trastuzumab and capecitabine [45]. Currently, its use is primarily reserved for third-line treatment. Emerging real-world data, which includes patients up to 85 years of age, indicates encouraging efficacy results even following progression on T-DXd, although the patient numbers remain limited [46].

In the HER2CLIMB study, among the 480 patients evaluated for PFS, no significant differences in efficacy were observed between patients ≥ 65 years (51 patients; HR 0.59; 95% CI 0.32–1.11) and younger patients (224 patients; HR 0.54; 95% CI 0.41–0.82). There were also no differences in OS, with an HR of 0.58 (95% CI 0.32–1.06) in 53 patients ≥ 65 years and an HR of 0.69 (95% CI 0.5–0.95) in 162 patients < 65 years. In those patients ≥ 65 years with central nervous system metastases, the benefit was comparable although their representation was small [47, 48]. In this study, 34% of patients ≥ 65 years experienced severe adverse events (diarrhea and vomiting) and treatment interruption, compared to 28% of those < 65 years. Since the population of patients ≥ 75 years was small, safety in this subgroup could not be fully evaluated.

According to pharmacokinetic analyses, age does not have a clinically significant influence on the absorption of tucatinib, so dose adjustment is not recommended in older patients. However, tucatinib has not been investigated in patients over 80 years of age [49].

According to these results, tucatinib in combination with trastuzumab and capecitabine is recommended as third line of treatment after T-DXd in elderly fit patients (III-B).

#### 4.2.3 Trastuzumab emtansine

In the EMILIA study, which included patients with HER2-positive metastatic breast cancer previously treated with trastuzumab and a taxane, T-DM1 demonstrated a benefit compared to lapatinib plus capecitabine in both PFS (9.6 months with T-DM1 *vs*. 6.4 months with lapatinib plus capecitabine; HR 0.65; 95% CI 0.55–0.77; *p* < 0.001) and OS (30.9 months with T-DM1 *vs.* 25.1 months with lapatinib plus capecitabine; HR 0.68; 95% CI 0.55–0.85; *p* < 0.001) [50]. The median age of the patients was 53 years (24–84 years), including 113 patients between 65 and 74 years, and 25 patients ≥ 75 years. This benefit was consistently observed across all subgroups, with a smaller benefit among older patients and those with non-visceral or non-measurable diseases (Table 3) [50].

**Table 3:** First and subsequent lines of treatment for advanced HER2-positive breast cancer

Study	Patients	Treatment	Results
*First-line treatment*			
CLEOPATRA Phase III NCT00567190 [33]	n = 808 16% ≥ 65 yr	A: P + H + T B: Placebo + H + T	A *vs.* B PFS: 18.7 *vs.* 12.4 months; *p* < 0.001 OS: 56.5 *vs.* 40.8 months; *p* < 0.001
Dang et al. [34]	n = 69 53 yr (26–84)	Tx + H + P	PFS: 19.5 months
PERTAIN Phase II NCT01491737 [35]	n = 258 33% ≥ 65 yr 19% ≥ 75 yr	A: P + H + AI B: H + AI	A *vs.* B PFS: 18.9 *vs.* 15.8 months; *p* = 0.007 ≥ 65 yr, HR 0.66; 95% CI 0.39–1.12 ≥ 75 yr, HR 0.65; 95% CI 0.31–1.35
HERNATA Phase III NCT00430001 [36]	n = 284 56 yr (29–72)	A: T + H B: V + H	A *vs.* B TTP: 12.4 *vs.* 15.3 months; *p* = 0.67 OS: 35.7 *vs.* 38.8 months; *p* = 0.98
VELVET Phase II, Cohort 1 NCT01565083 [37]	n = 106 56 yr (30–82)	P + H + V	ORR: 74% (95% CI 63.8–82.9) PFS: 14.3 months (95% CI 11.2–17.5)
EORTC 75111–10,114 trial NCT01597414 [10]	n = 80 ≥ 60 yr	A: P + H B: P + H + C	A *vs.* B 6-month PFS: 46% *vs.* 73%; *p* = 0.12 1-yr OS: 67% *vs.* 84%; *p* = 0.83
*Subsequent lines treatment*			
Krop et al. [39]	n = 851 21% ≥ 65 yr	T-DXd	DESTINY-Breast01; Breast02 and Breast03 ≥ 65 yr PFS: 19.4; 16.8 and 25.1 months ≥ 65 yr OS: 30.9; 30.2 and NR months
DESTINY-Breast03 Phase III NCT03529110 [40]	n = 524 22% ≥ 65 yr 3% ≥ 75 yr	A: T-DXd B: T-DM1	A *vs.* B 1-yr PFS: 76% *vs.* 34%; *p* < 0.001 < 65 *vs.* ≥ 65 yr ORR: 79% *vs.* 78%; PFS: 30 *vs.* 25 months
HER2CLIMB Phase II NCT02614794 [47]	n = 612 19% ≥ 65 yr	A: Tucatinib + H + X B: Placebo + H + X	A *vs.* B 1-yr PFS: 33% *vs.* 12%; *p* < 0.001 HR 0.54 (< 65 yr) *vs.* HR 0.59 (≥ 65 yr) 2-yr OS: 45% *vs.* 27%; *p* = 0.005 HR 0.69 (< 65 yr) *vs.* HR 0.58 (≥ 65 yr)
EMILIA Phase III NCT00829166 [50]	n = 991 11% 65–74 yr 3% ≥ 75 yr	A: T-DM1 B: Lapatinib + X	A *vs.* B PFS: 9.6 *vs.* 6.4 months; *p* < 0.001 HR 0.88 (65–74 yr) *vs.* HR 3.51 (≥ 75 yr) OS: 30.9 *vs.* 25.1 months; *p* < 0.001 HR 0.74 (65–74 yr) *vs.* HR 3.45 (≥ 75 yr)
TH3RESA Phase III NCT01419197 [51]	n = 602 15% ≥ 65 yr 3% ≥ 75 yr	A: T-DM1 B: Physician’s choice	A *vs.* B OS: 22.7 *vs.* 15.8 months; *p* < 0.001 < 65 *vs.* 65–74 *vs.* ≥ 75 years OS: 23.1 *vs.* 18.2 *vs.* 31.8 months
Cil et. al. [52]	n = 93 ≥ 65 yr	TDM-1	PFS: 8.5 months OS: 15.0 months
Geyer et al NCT00078572 [53]	n = 324	A: Lapatinib + X B: X	A *vs.* B PFS: HR 0.57; 95% CI 0.43–0.77; *p* < 0.001 TTP: 8.4 *vs.* 4.4 months; *p* < 0.001
Cetin et al. [54]	n = 26 ≥ 65 yr	Lapatinib + X	PFS: 7.0 months OS: 15.0 months
EGF104900 Phase III NCT00320385 [55]	n = 296 51 yr (26–81)	A: Lapatinib B: Lapatinib + H	A *vs.* B PFS: 8.1 *vs.* 12.0 weeks; *p* = 0.008 OS: 39.0 *vs.* 51.6 weeks; *p* = 0.106
NALA Phase III NCT01808573 [56]	n = 621 21% ≥ 65 yr	A: Neratinib + X B: Lapatinib + X	A *vs.* B PFS: 8.8 *vs.* 6.6 months; *p* = 0.006 HR 0.74 (< 65 yr) *vs.* HR 0.83 (≥ 65 yr) OS: 24.0 *vs.* 22.2 months; *p* = 0.209 HR 0.86 (< 65 yr) *vs.* HR 0.86 (≥ 65 yr)

AI: aromatase inhibitor; AEs: adverse events; C: cyclophosphamide; CI: confidence interval; H: trastuzumab (Herceptin®); HR: hazard ratio; C: cyclophosphamide; NR: not reported; ORR: objective response rate; OS: overall survival; PFS: progression-free survival; T: docetaxel (Taxotere®); T-DM1: trastuzumab emtansine; T-DXd: trastuzumab deruxtecan; TTP: time to progression; Tx: paclitaxel (Taxol®); V: vinorelbine; X: capecitabine (Xeloda®); yr: years

In a sub-analysis of patients from the KAMILLA study conducted to investigate the safety profile of T-DM1 in patients older and younger than 65 years (884 patients in total; 122 [14%] ≥ 65 years), it was confirmed that there were more grade 3–4 adverse events in older patients, who also had more treatment interruptions as a result [57].

In patients who had progressed after a taxane, lapatinib, and trastuzumab, the TH3RESA study evaluated the efficacy of T-DM1 *vs.* treatment of the investigator´s choice (Ib-A) [51]. Fifteen percent of patients were ≥ 65 years and 3% were ≥ 75 years. A pre-specified OS analysis by age subgroups (< 65 *vs.* 65–74 *vs.* ≥ 75) showed a median OS of 23.1 months (HR 0.71; 95% CI 0.55–0.91), 18.2 months (HR 0.73; 95% CI 0.40–1.34), and 31.8 months (HR 0.27; 95% CI 0.07–1.04), respectively.

Recently, in a real-world clinical data study, both the safety and efficacy of TDM-1 were investigated in 93 patients ≥ 65 years (III-B) [52]. The median PFS and OS were 8.5 and 15.0 months, respectively. Adverse events were observed in 93% of the patients, with grade 3–4 adverse events occurring in 30% of them. The authors concluded that the efficacy of T-DM1 was acceptable and, in general, well-tolerated among elderly patients.

Based on this information, we recommend the use of T-DM1 in elderly patients as it has demonstrated an acceptable efficacy and, in general, good tolerance among elderly patients (Ib-A). However, it must consider the small sample sizes of the studies, the heterogeneity of the populations included as well as retrospective nature of some of them.

#### 4.2.4 Lapatinib plus capecitabine

The efficacy of the combination of lapatinib with capecitabine was evaluated in a randomized phase III study published by Geyer et al. in 2006 [53]. The study included patients with HER2-positive breast cancer who had progressed after prior treatment with anthracyclines, taxanes, and trastuzumab. The combination of lapatinib and capecitabine, compared to a capecitabine monotherapy, showed a 51% reduction in time to disease progression (HR 0.49; 95% CI 0.34–0.71; *p* < 0.001) and a median time to progression of 8.4 vs 4.4 months, respectively. However, efficacy and safety data by age subgroup were not available. A retrospective analysis of a series of 26 patients aged ≥ 65 years treated with lapatinib plus capecitabine showed a median PFS of 7.0 months, which is close to the 8.4 months of time to progression observed in the 2006 phase III study [54].

Considering this information, we recommend using the combination of lapatinib with capecitabine with caution, as we only have retrospective data on the efficacy and tolerability of this schedule in a small group of patients aged ≥ 65 years (IV-C).

#### 4.2.5 Neratinib plus capecitabine

The NALA study included 129 (21%) patients ≥ 65 years and compared the efficacy of capecitabine in combination with neratinib *vs*. lapatinib. The combination with neratinib significantly improved PFS compared to the combination with lapatinib (8.8 *vs.* 6.6 months; HR 0.76; 95% CI 0.63–0.93; *p* = 0.006), with the benefit maintained both in patients < 65 years (HR 0.74; 95% CI 0.60–0.91) and those ≥ 65 years (HR 0.83; 95% CI 0.53–1.28). However, in terms of OS, the combination with neratinib did not show a significant benefit compared to the combination with lapatinib (24.0 *vs.* 22.2 months; HR 0.88; 95% CI 0.72–1.07; *p* = 0.209) [56].

In 2021, a phase II study on the safety and tolerability of neratinib in 25 patients aged ≥ 60 years (range 60–79) without limitation on the number of prior HER2-targeted therapies (mean 3, range 0–11) was published [58]. Neratinib was shown to be safe in this group, with grade ≥ 2 toxicities in 80% of the patients and grade 3 toxicities in 36% of the patients, which included diarrhea (20%), abdominal pain (20%), and vomiting (8%). No grade 4 or 5 toxicities were observed. In Spain, the use of neratinib for metastatic patients is not funded by the National Health System [59].

## Toxicity management in the proposed treatments

Optimizing the management of toxicity associated with anti-HER2 treatment in elderly patients is crucial. These therapies, whether as monotherapy or combined with chemotherapy, present a range of side effects. Cardiac dysfunction remains the most significant across all treatments, while gastrointestinal issues, hematologic abnormalities and infusion-related reactions are more commonly observed when combined with chemotherapy [60]. Older patients are particularly vulnerable to these toxicities due to physiological changes associated with age, comorbidities, and altered drug metabolism [61].

Cardiotoxicity is among the most significant complications of anti-HER2 therapies, with trastuzumab being one of the most studied agents in this regard. It primarily manifests as an asymptomatic reduction in the left ventricular ejection fraction (LVEF), occurring in approximately 10% of cases. In a smaller proportion of cases, it may progress to symptomatic heart failure, with incidence varying from 1 to 4% according to clinical trials involving selected patients [62, 63].

It is important to note that, unlike anthracycline-induced cardiotoxicity, trastuzumab-related cardiotoxicity does not appear to be directly related to cumulative drug dose. Moreover, it is often reversible upon treatment interruption, allowing reintroduction once cardiac function has recovered. This difference in the toxicity profile suggests that trastuzumab-associated cardiac dysfunction might be linked to an alteration of the myocardial contractility rather than to myocyte death [64]. Establishing a cardio-oncology unit is recommended to optimize risk factor control and cardiovascular disease management, implement protective therapeutic measures, and identify and promptly treat potential cardiovascular toxicities. This approach could lead to a reduction in discontinuation rates of anti-HER2 therapy, potentially impacting survival outcomes and optimizing survivorship follow-up [65].

The incidence of cardiotoxicity in patients > 65 years old is notably higher than in younger populations, reaching up to 20–30% according to various retrospective studies [66–70], especially among those with a history of hypertension, diabetes, obesity, baseline LVEF < 55%, or prior/current use of anthracyclines. Typically, it manifests within the first year of treatment, although most cases can be managed on an outpatient basis, underscoring the importance of cardiac monitoring for early detection and optimal management of this complication.

While predictive biomarkers, such as proBNP and troponin, have been investigated, and risk scales considering age and baseline LVEF have been developed, their utility in clinical practice remains undetermined and has not been validated in the general population [71, 72]. Nevertheless, despite the absence of specific guidelines for management, certain recommendations based on expert opinions and defined trial protocols can be followed [73]. These include: (i) perform a baseline cardiac assessment with echocardiography or radioisotope angiography before initiating trastuzumab treatment; (ii) perform cardiac evaluations every 3 months during treatment and every 6 months post-treatment discontinuation up to 24 months from the last adjuvant trastuzumab administration; (iii) for metastatic treatment, a cardiac reassessment should be made if symptoms of heart failure appear; (iv) in patients at higher risk of cardiotoxicity, such as those with baseline LVEF < 55% or hypertension, referral to cardiology for optimizing treatment with angiotensin-converting enzyme inhibitors or beta-blockers should be considered. Although several clinical trials have investigated the efficacy of these treatments, the results remain inconclusive [74].

If a patient develops cancer therapy-related cardiac dysfunction (CTRCD) due to anti-HER2 treatment, it is recommended to follow the guidelines outlined in the 2022 Cardio-Oncology Guide, developed in collaboration with the European Haematology Association (EHA), the European Society for Therapeutic Radiology and Oncology (ESTRO), and the International Cardio-Oncology Society (IC-OS) as summarized in Table 4.

**Table 4:** Management of patients with anti-HER2 therapy-related CTRCD [73]

Risk	Description	Oncological treatment	Cardiovascular treatment
*Symptomatic CTRCD*			
Severe to moderate	Hospitalization or intensification of cardiac treatment is required	Interruption of anti-HER2 therapy	Optimization of HF treatment
Mild	Intensification of cardiac treatment is not required	Multidisciplinary assessment to continue or interrupt anti-HER2 therapy	Maintenance of HF treatment
*Asymptomatic CTRCD*			
Severe	LVEF < 40%	Interruption of anti-HER2 therapy and multidisciplinary evaluation for possible treatment reintroduction with LVEF ≥ 40%	Initiation of HF treatment
Moderate	LVEF 40–49%	Continuation of anti-HER2 therapy with closer cardiovascular monitoring	HF treatment with cardio-protective therapy (ACE-I / ARB and BB)
Mild	LVEF > 50% GLS decrease > 15% Cardiac biomarker increase	Continuation of anti-HER2 therapy with closer cardiovascular monitoring	HF treatment with cardio-protective therapy (ACE-I / ARB and BB)

ACE-I: angiotensin-converting enzyme inhibitors; ARB: angiotensin receptor blockers; BB: beta-blockers; CTRCD: cancer therapy-related cardiac dysfunction; GLS: global longitudinal strain; HF: heart failure; LVEF: left ventricular ejection fraction

These recommendations can be extrapolated to other anti-HER2 therapies, although cardiac toxicity varies depending on the type of anti-HER2 therapy: (i) trastuzumab has a cardiotoxicity incidence of 7%, rising to 27% when combined with anthracyclines and 13% when combined with paclitaxel, manifesting left ventricular dysfunction and other cardiac events; (ii) pertuzumab has a low risk of cardiotoxicity but can double the risk of heart failure in some cases; (iii) lapatinib has a cardiotoxicity of 1.6%, mostly reversible, with only 0.2% of cases with symptomatic heart failure; (iv) T-DM1 has an incidence of left ventricular dysfunction of 1.7%, comparable to the combination of lapatinib and capecitabine (1.6%) [75]. The low rates of cardiotoxicity observed with T-DM1 are comparable to those reported with T-DXd. However, in the pivotal trials, patients had to have an LVEF ≥ 50% to be eligible. Therefore, the rates of cardiotoxicity reported in these studies may not represent real-life scenarios [40, 50, 51].

It is important to consider other toxicities, such as gastrointestinal and infusion reactions. These may require additional supportive measures, such as the use of antidiarrheal agents, antiemetics, and dietary modifications, to minimize the impact of toxicity on the patient´s quality of life and ensure the continuity of the oncological treatment.

## Main clinical guidelines recommendations

There are no specific recommendations for the treatment of elderly patients (≥ 70 years) with HER2-positive breast cancer in any national, European, or American guideline [76–79]. The ESMO guidelines for early breast cancer recommend adapting complementary treatment to the biological, not chronological, situation of each patient. For patients eligible for chemotherapy, it is recommended, opting for less toxic regimens in frail patients [76].

The 2021 consensus guidelines by EUSOMA and SIOG recommend tailoring treatment based on the risk of toxicity [80]. Therefore, in early breast cancer, it is advisable to prioritize regimens without anthracyclines, to add pertuzumab in high-risk fit patients, to avoid extended treatment with neratinib due to the risk of severe diarrhea, and to consider shorter duration treatments with trastuzumab in small tumors [81].

In metastatic breast cancer, unless there is a cardiac contraindication, the treatment would still involve dual anti-HER2 blockade combined with chemotherapy, prioritizing paclitaxel, or with vinorelbine, capecitabine, or metronomic cyclophosphamide in patients not eligible for taxanes. In cases of hormone receptor-positive and frail patients, a continuation with hormonal treatment could be considered [80].

## Conclusions

The management of breast cancer in elderly patients is a challenging but extremely important task for the medical team. In the era of an aging population, clinical decisions must be optimized based on various factors beyond the patient´s age. These factors include comorbidities, functional status, life expectancy, pathological and molecular characteristics of the tumor, and the patient´s wishes. A complete CGA, a process that should always be performed, is crucial to avoid insufficient or unnecessary treatments or exposing patients to non-tolerable toxicities.

Although anti-HER2 therapies, such as trastuzumab and its combination with pertuzumab and chemotherapy, are effective and generally well-tolerated in elderly patients, they have a characteristic toxicity profile, especially concerning cardiac toxicity. Shorter trastuzumab-based schedules (6 months), regimens with limited cardiac toxicity or a chemotherapy-free strategy may be warranted, especially in vulnerable patients, The neoadjuvant approach for HER2 + tumors has gained attention, but its use in older patients should be considered with caution. Due to the increased risk of side effects and the possibility that older patients may be in poorer condition for surgery, it is important to consider their overall health before proceeding with this approach. Thus, neoadjuvant treatment should be reserved primarily for fit and high-risk older patients. Trastuzumab remains the foundation of anti-HER2 treatment, and its combination with pertuzumab and chemotherapy has shown significant benefits. However, specific evidence in patients over 65 years is limited.

For the treatment of advanced stages, the standard recommendation remains a dual HER2 blockade strategy (trastuzumab and pertuzumab) with chemotherapy as first line. However, the choice of treatment regimen must be carefully adapted to each patient´s condition, considering their overall health status and comorbidities. The combination of trastuzumab and pertuzumab with paclitaxel has demonstrated good safety and efficacy in elderly patients.

In successive lines of treatment, T-DXd emerged as an effective option after progression with previous treatments, demonstrating its efficacy in older patients. Tucatinib, in combination with capecitabine and trastuzumab, also provides significant benefits in PFS and OS with a manageable toxicity profile. Including elderly patients in clinical trials is important to obtain more accurate data on the efficacy and safety of these treatments in this population.

Proper management of toxicity (especially cardiotoxicity) and continuous monitoring are essential to improve outcomes and quality of life in older patients with HER2-positive breast cancer. We recommend close cardiac monitoring for elderly patients undergoing anti-HER2 therapy, including baseline assessments with echocardiography followed by evaluations every 3 months during treatment. In patients at higher risk of cardiac events, such as those with baseline low ejection fraction or significant comorbidities, more frequent monitoring and consultation with cardio-oncology specialists are advised. Additionally, managing gastrointestinal and hematologic toxicities requires proactive interventions, including hydration protocols and nutritional support as necessary to maintain quality of life.

It is imperative to continue research and adapt clinical guidelines to better meet the needs of this growing population, ensuring effective and safe treatments. Furthermore, more clinical trials or real-world evidence studies are required to address the actual tolerability and efficacy of treatments, taking into account the comorbidities and frailty of older patients.

## Data Availability

Not applicable.
